# Case report: incidental findings of COVID-19 infection on positron emission tomography/computed tomography for staging of a giant gastric gastrointestinal stromal tumor

**DOI:** 10.11604/pamj.supp.2020.35.2.23167

**Published:** 2020-05-11

**Authors:** Hamish Reed-Embleton, Khurram Shahzad Khan, Navin Mathias, Sajid Mahmud

**Affiliations:** 1 Department of Surgery, University Hospital Hairmyres, East Kilbride, Scotland, UK; 2 Department of Radiology, University Hospital Hairmyres, East Kilbride, Scotland, UK

**Keywords:** Covid-19, FDG-PET, GIST, gastrointestinal stromal tumor

## Abstract

We report the incidental finding of COVID-19 in a 59-year-old male, with no significant cardiorespiratory past medical history who underwent a fluorodeoxyglucose positron emission tomography (FDG-PET) scan for investigation of a likely gastric gastrointestinal stromal tumor (GIST). There may be significant discrepancies between clinical symptoms and radiological severity with COVID-19 infection. FDG-PET scanning has the potential to complement traditional radiological imaging in COVID-19 in diagnosis of subclinical diagnosis or early stage disease, as well as monitoring disease progression.

## Introduction

COVID-19 was first reported in Wuhan, Hubei province, China in December 2019 [1]. In the UK, the first case of COVID-19 was reported on 31st January 2020. There are over 150,000 confirmed cases in the UK with over 20,000 reported deaths by the end of April 2020 [2]. COVID-19 symptoms can be non-specific ranging from no symptoms to severe pneumonia and death. RT-PCR remains the gold standard investigation but has high false negative rates [3]. Plain chest X-ray films may also demonstrate normal findings in the early stages of infection and are not sensitive in the detection of ground glass opacities [4]. This report emphasises the need to remain vigilant as asymptomatic carriers of COVID-19 may undergo nuclear medicine investigation.

## Patient and observation

**Figure 1:**
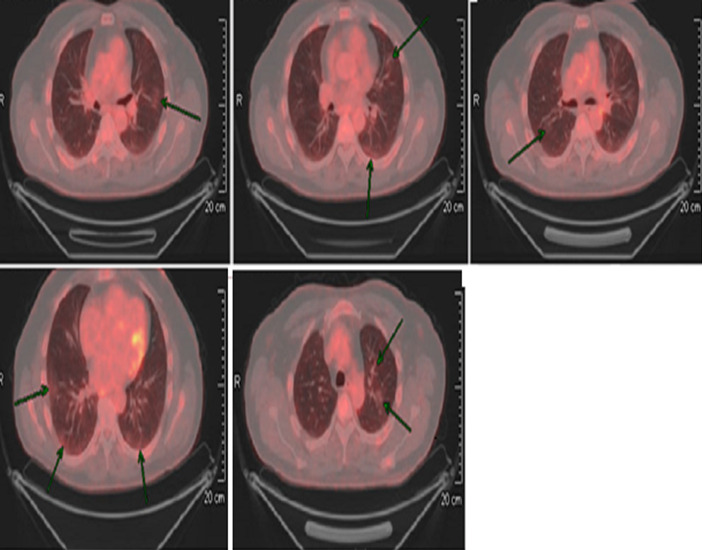
CT PET showing faintly FDG-avid patchy prominently peripheral ground-glass changes in bilateral upper and lower lobes in keeping with probable COVID-19 infection

A 59-year-old male with no cardiorespiratory or other co-morbidities was referred for investigation of intermittent dysphagia. He underwent upper gastrointestinal (GI) endoscopy. This showed possible gastrointestinal stromal tumor (GIST) in the proximal stomach, although biopsies on two occasions showed normal gastric mucosa. A computed tomography (CT) scan performed in March 2020 which showed a heterogenous mass intimately connected to the gastric fundus, suggestive of gastric GIST and measuring 15 x 12.6cm. Discussion within a regional sarcoma multidisciplinary team suggested referral for endoscopic ultrasound (EUS) and functional imaging with fluorodeoxyglucose positron emission tomography (FDG-PET). The FDG-PET scan was performed four weeks later. At this stage he remained apyrexial (body temperature < 37.50C), with no respiratory or gastrointestinal symptoms, or any headache and there was no history of exposure to possible COVID-19. The FDG-PET scan confirmed the CT findings and reported no significant change or size in the GIST but showed FDG-avid, prominently peripheral, ground-glass changes in bilateral upper and lower lobes (Figure 1). The changes were new compared with the CT dated 20th March 2020 and were in keeping with probable COVID-19 infection [1,5,6]. His FDG-PET scan results were discussed via phone call and he was advised to self-isolate. He remains asymptomatic.

## Discussion

Our case again highlights the speed of disease progression and dramatic radiological findings despite the apparent lack of symptoms in keeping with recent data that suggests up to 78% of patients with a COVID-19 infection may be asymptomatic [7,8]. Viral infection causes an inflammatory cascade during which all activated leucocytes are dependent on glucose for anaerobic glycolysis which causes a high FDG uptake during FDG-PET scanning [3,9]. Qin et al., reported FDG-PET scans of four confirmed or suspected COVID-19 patients [10]. All lesions showed high trace uptake (SUVmax 1.8-12.2), demonstrating the high inflammatory burden associated with COVID-19 infection and noted that patients with higher FDG uptake took longer to heal [10]. This supports the potential use of FDG-PET scanning to aid in determining the progression, and outcomes in patients with COVID-19.

Chefer et al. reported the ability of FDG-PET to detect even subtle changes with subclinical MERS-CoV infection in 2015 [11]. Such findings indicate the potential added value of FDG-PET imaging when presentation, is non-specific or atypical. Interestingly FDG-PET scanning is an important tool for investigating fever of unknown origin and measuring the inflammatory response to infection [9]. Radiologic imaging techniques such as CT scan are able to detect focal inflammatory and infectious processes. However, during the early phase of infection, the anatomical change may be insubstantial for their detection by CT scan compared with FDG-PET scan [9]. This is supported by Bellani et al., who reported, in patients with acute respiratory distress syndrome (ARDS), FDG-PET imaging has been shown to identify the increase metabolic rates of pulmonary tissues when CT scanning finds them to be normal [12]. FDG-PET scanning has further been shown to aid in the prediction of patients developing ARDS following thoracic trauma and pulmonary contusion [13].

## Conclusion

With a relatively low radiation exposure, radionucleotide imaging may be able to give a more complete picture of acute respiratory viral infections and the development of pulmonary parenchymal injury and may enable more logical decision making around use of specific interventions such as the use of anti-inflammatories [14,15]. Therefore, it has the potential to complement CT scan and other imaging modalities in identifying the pulmonary structural changes, evaluating infectious and immune phases and monitoring disease progression during the course of acute respiratory illness with COVID-19 infection [14,16]. However, in the current era of financial strain this test is not feasible or readily available for diagnosing COVID-19.

## Competing interests

The authors declare no competing interests.
